# SUSHI: an exquisite recipe for fully documented, reproducible and reusable NGS data analysis

**DOI:** 10.1186/s12859-016-1104-8

**Published:** 2016-06-02

**Authors:** Masaomi Hatakeyama, Lennart Opitz, Giancarlo Russo, Weihong Qi, Ralph Schlapbach, Hubert Rehrauer

**Affiliations:** Functional Genomics Center Zurich, ETH Zurich/University of Zurich, Winterthurerstrasse. 190, 8057 Zurich, Switzerland; Department of Evolutionary Biology and Environmental Studies, University of Zurich, Winterthurerstrasse. 190, 8057 Zurich, Switzerland

**Keywords:** Data analysis framework, Reproducible research, Meta-level system design

## Abstract

**Background:**

Next generation sequencing (NGS) produces massive datasets consisting of billions of reads and up to thousands of samples. Subsequent bioinformatic analysis is typically done with the help of open source tools, where each application performs a single step towards the final result. This situation leaves the bioinformaticians with the tasks to combine the tools, manage the data files and meta-information, document the analysis, and ensure reproducibility.

**Results:**

We present SUSHI, an agile data analysis framework that relieves bioinformaticians from the administrative challenges of their data analysis. SUSHI lets users build reproducible data analysis workflows from individual applications and manages the input data, the parameters, meta-information with user-driven semantics, and the job scripts. As distinguishing features, SUSHI provides an expert command line interface as well as a convenient web interface to run bioinformatics tools. SUSHI datasets are self-contained and self-documented on the file system. This makes them fully reproducible and ready to be shared. With the associated meta-information being formatted as plain text tables, the datasets can be readily further analyzed and interpreted outside SUSHI.

**Conclusion:**

SUSHI provides an exquisite recipe for analysing NGS data. By following the SUSHI recipe, SUSHI makes data analysis straightforward and takes care of documentation and administration tasks. Thus, the user can fully dedicate his time to the analysis itself. SUSHI is suitable for use by bioinformaticians as well as life science researchers. It is targeted for, but by no means constrained to, NGS data analysis. Our SUSHI instance is in productive use and has served as data analysis interface for more than 1000 data analysis projects. SUSHI source code as well as a demo server are freely available.

**Electronic supplementary material:**

The online version of this article (doi:10.1186/s12859-016-1104-8) contains supplementary material, which is available to authorized users.

## Background

Today’s bioinformatics faces the practical challenge to analyze massive and diverse data in a well documented and reproducible fashion. The situation is particularly challenging in the area of NGS research where state-of-the-art algorithms are frequently available as standalone tools and where a complete data analysis consists of many individual data processing and analysis steps. The considerations associated with conducting such a data analysis in a research environment have been discussed by W. S. Noble [1] and guidelines as well as an example strategy for organizing computational data analysis have been given. According to Noble a key principle is to record every operation such that reproducibility is ensured.

In this paper, we present SUSHI, which does Support Users for SHell-script Integration, a new approach to bioinformatics analysis that is centered on reusability, reproducibility and scalability. SUSHI produces analysis results as directories that are fully self-contained and hold all the information to be reproduced. Specifically, we document all parameters, input data, commands executed, as well as the versions of the tools and the reference data used. Additionally, we store meta-information on the experimental data together with the result files, so that those can be interpreted and further analyzed by other tools independently from SUSHI. This holds even if the analysis directory is transferred to collaborators with a different computing environment. SUSHI is extendable and we have put special emphasis on the simplicity of adding new software applications. A bioinformatician can define them within a single file and does not need special programming skills. SUSHI natively offers a command line interface as well as a web interface to run data analysis steps. Altogether, SUSHI lets bioinformaticians efficiently build analysis pipelines and ensures that analysis results are ready-to-be-shared and reproducible.

Various types of data analysis frameworks have already been implemented. They can be essentially divided into web-based frameworks and scripting frameworks. Examples for web-based frameworks are Galaxy [2], Chipster [3], GeneProf [4] or GenePattern [5]. They let users run individual steps or entire pipelines on a remote compute system with the framework keeping track of the executed analysis. Scripting frameworks like bpipe [6], Ruffus [7], nestly [8], NGSANE [9], Makeflow [10], and Snakemake [11], let users build bioinformatics pipelines in a command line fashion. Given the different types of user interactions, the former solutions are more targeted for the experienced biologists or the application-oriented bioinformaticians while the latter address the needs of bioinformaticians who are more inclined to programming and high-throughput analysis of many datasets. However, there is no system as yet that natively offers both interfaces. Additionally, none of the existing frameworks puts an emphasis on having a human-readable and portable file-based representation of the meta-information and associated data.

## Implementation

### SUSHI data sets and applications

**Fig. 1:**
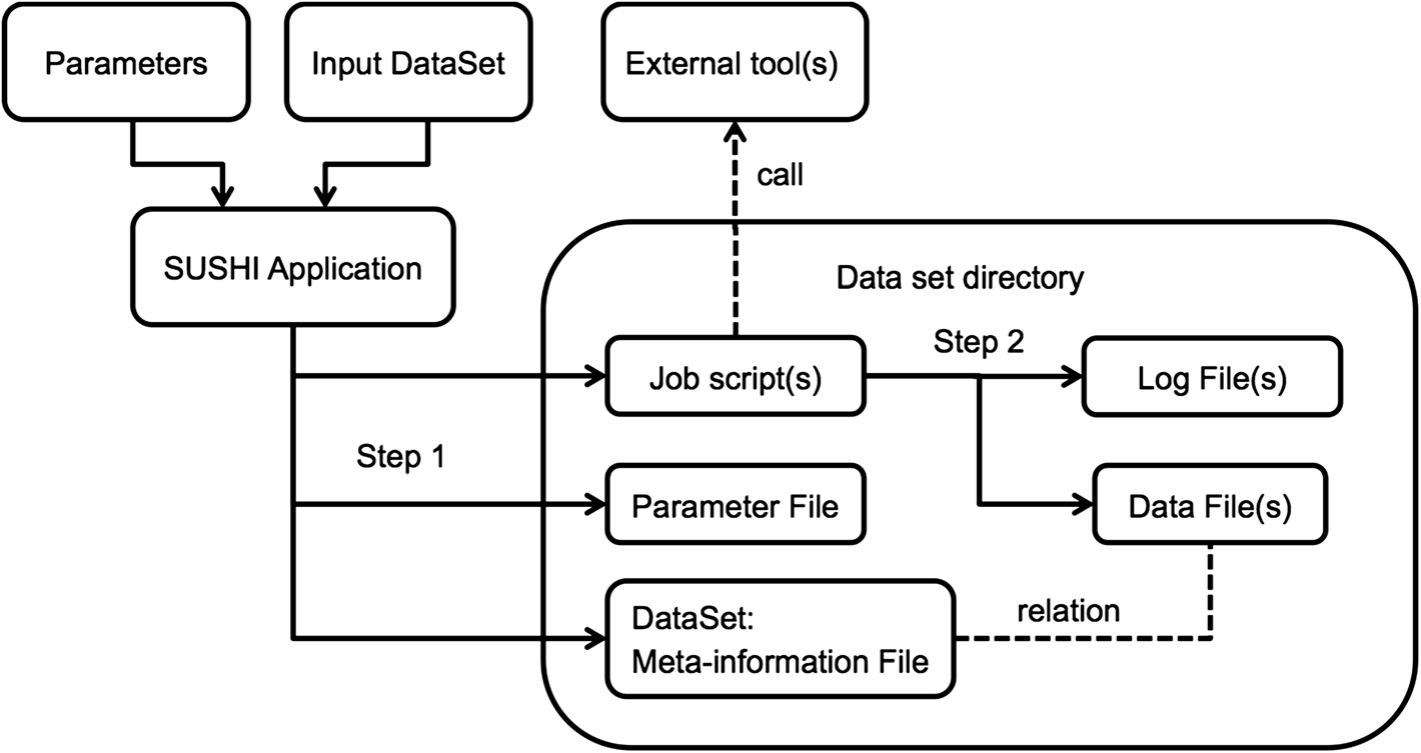
The use case of DataSet generation. By running a SUSHI application with an input DataSet and parameters, a new DataSet is generated. Initially (Step 1) only the meta-information, the parameter file, and the job scripts are generated. The actual data files and the log files are generated by executing the static job scripts (Step 2)

Within SUSHI, the original measured data as well as any derived analysis result is modeled as a data set that is represented as a fully self-sufficient directory on the file system. For the original data this means that it must be accompanied by meta-information on how the data has been measured. The meta-information must include information on the biological samples used as well as information on the measurement process. For analysis results this implies that the analysis result files must have accompanying meta-information that documents the input data, the analysis steps, and the analysis parameters. If these requirements are satisfied, we call this a *dataset*. Figure 1 shows schematically how a dataset is generated as the result of running a SUSHI application. The meta-information associated with a dataset is represented in SUSHI as a DataSet object. On the file system a tabular file called *dataset.tsv* represents the dataset. Examples of meta-information are characteristics like sample name, species, tissue, but also e.g. the genome build that has been used for read alignment. Each characteristic is represented as a separate column in the tabular dataset.tsv.

A SUSHI application requires as input both a set of parameters and a DataSet object. This means that applications do not take bare data files as direct input. Instead, SUSHI applications take as input the DataSet meta-information object. The DataSet object holds, next to the data files, the meta-information necessary to process and interpret the data files. Based on its input, a SUSHI application first generates 1) the necessary job script(s), 2) a file representation of the parameters, and 3) the DataSet for the output data (Fig. 1 Step 1). The actual result data file(s) are generated by the job script(s) (Fig. 1 Step 2). The columns of the output DataSet hold again the meta-information, which now include additionally the parameters of the executed analysis if relevant for the further analysis or interpretation. The set of characteristics that is added to the annotation columns is defined and generated by the SUSHI application. The SUSHI framework itself does not require any specific annotation columns. Thus, the semantics of the DataSet columns are determined by the SUSHI applications (described in detail in the next section).

Every column of meta-information has a unique header that identifies the content, and optional tags that characterize the information type in the column. Tags are represented as comma-separated strings within square brackets in the column headers. Currently supported tags are *File*, *Link*, *Factor*, and *Characteristics*. Depending on the tags, the SUSHI framework provides appropriate actions for the corresponding columns. The File tag is reserved for actual file paths, and SUSHI checks if the file actually exists. If a column has a Link tag, SUSHI will automatically add a hyperlink to the data. Finally, the Factor column data will be used to group samples according to experimental factors, which is typically required in a differential gene expression analysis.

### Example DataSet holding RNA-seq reads

We follow the convention that sequencing read files are represented as a DataSet with the following columns:

- Name: the name of the sample measured.
- Read1 [File]: path to the file holding the reads; if reads are paired-end, this must be the first read.
- Adapter1: potentially contaminating adapter sequence at the 3′-end of read 1.
- Read2 [File]: path to the second read for paired-end data (only for paired-end data).
- Adapter2: potentially contaminating adapter sequence at the 3′-end of read 2 (only for paired-end data).
- Species: the species of the sample.
- StrandMode: specifies whether the library preparation protocol preserved strand information.
- Enrichment Kit: the kit employed to enrich the input material (e.g. poly-A selection kit)
- Read Count: the number of reads in the file.

Additionally, there can be columns that specify experimental factors. Table 1 shows an example (due to space constraints only a subset of the columns is shown).

**Table 1:** A sample DataSet

Name	Read [File]	Species	Genotype [Factor]
Mut1	P1001/ventricles/mut1_R1.fastq.gz	Mus musculus	Mutant
Mut2	P1001/ventricles/mut2_R1.fastq.gz	Mus musculus	Mutant
Wt1	P1001/ventricles/wt1_R1.fastq.gz	Mus musculus	Wildtype
Wt2	P1001/ventricles/wt2_R1.fastq.gz	Mus musculus	Wildtype

Example of a sequencing read DataSet where a subset of the meta-information is shown as annotation columns. The DataSet includes four samples with four categories of meta-information, 1. Name, 2. Read, 3. Species, and 4. Genotype. Each column header can have a tag. E.g. *[File]* means the column holds file locations, and *[Factor]* means the values represent an experimental factor. The DataSet object is implemented as an Array of Hash objects in the SUSHI system and it can be imported from or exported to tab-separated-value file

**Fig. 2:**
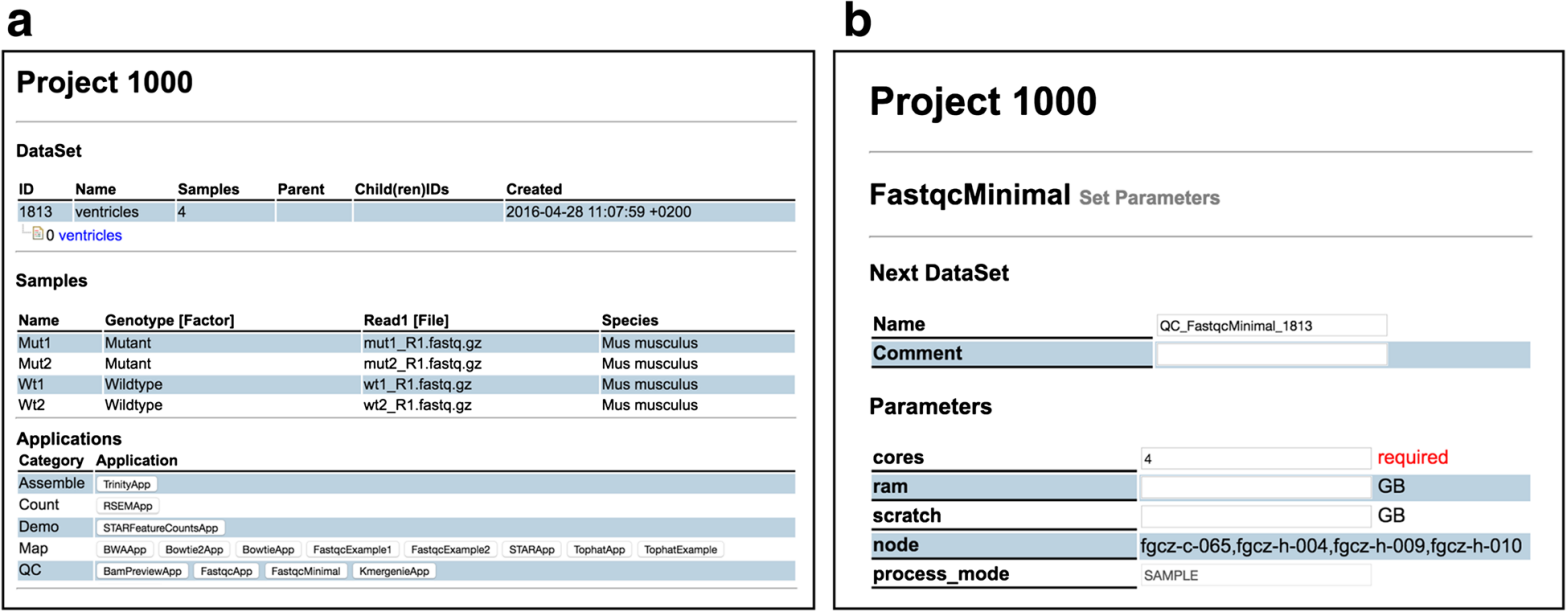
The screenshots of a DataSet and parameter setting view. **a** DataSet view shows basic information of the DataSet, sample information, and the compatible SUSHI applications at the bottom. The SUSHI application is shown as a button and categorized based on the *@analysis_category* defined in the SUSHI application Ruby code. **b** After selecting a SUSHI application, the parameter setting view lets users modify the analysis parameters. According to the SUSHI application definition, GUI components are auto-generated and placed in the view

It is important to mention here that the SUSHI framework does not impose any constraints or semantics on the columns of the DataSet table. It is up to the user to identify which meta-information is relevant for his data. In particular, we do not require specific ontologies or controlled vocabularies. Users are free to define their own meta-information. The content definition and interpretation is entirely delegated to the user and the SUSHI applications. Figure 2a shows how the above DataSet is visualized in the SUSHI DataSet view.

### Example SUSHI application performing a FastQC report

A common task is to generate a FastQC report [12] for each sample in a read data set. If the fastqc package is installed one would run e.g.

which creates the FastQC report mut1_R1_fastqc.zip for the first sample in the above data set. With the SUSHI framework this can be turned into a SUSHI application defined by the following Ruby code:
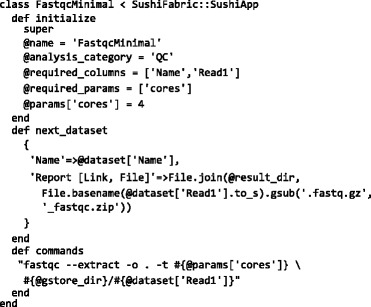


The example code shows the essential features of a SUSHI application (See also Additional file 1). The *@required_columns* tells the SUSHI framework which columns a DataSet must have so that *FastqcMinimal* is applicable. In Fig. 2a, all applications that are compatible with the example reads data set are shown at the bottom, including the *FastqcMinimal* application. The *@params['cores']* defines the number of cores to be used for multi-threading as a parameter with default value 4. This parameter is automatically turned into an input field in the web interface (see Fig. 2b. The code also defines with the method *next_dataset* the columns and content for the resulting DataSet. Finally, the method *commands* defines the command to be executed.

The SUSHI framework automatically performs administrative tasks such as putting the resulting file in the correct directory and managing the log files The full result directory is available as Additional file 2. A second example is the example for TopHat mapping [13] in Additional files 3 and 4. Both examples are for the illustrative purpose kept minimal. In real world application one would define additional parameters, support paired-end reads, and so on.

A list of all SUSHI applications that is in use at the Functional Genomics Center Zurich is available as Additional file 5 or on the SUSHI demo server: http://fgcz-sushi-demo.uzh.ch/sushi_application.

### SUSHI applications are meta-process objects

Conceptually, a SUSHI application is a meta-process that generates an actual application (shell script). The entire data analysis functionality is contained within the shell script. For example, for a read alignment application, the job script contains the call to the aligner. If a SUSHI application is suitable only for a certain data type, it defines requirements on the input data. The requirements are specified as mandatory DataSet columns that an input dataset must have. The SUSHI framework guarantees that applications are only available for compatible DataSets. At run time the SUSHI application defines the columns of the resulting DataSet it will produce. With this meta-process modeling approach, the applications define the semantics of the meta-information without referring to other applications but only to DataSet content. Finally, from the input data and the parameters provided, the core method of a SUSHI application builds the command lines as a shell script that will eventually produce the output. The shell script can call other scripts or tools on the execution server. The only constraints for tools are

- Must be runnable from command line.
- Must not require interactive input at run time.
- Result must be representable as a file, or a set of files in a directory.

The framework cares about job execution, data file placement and cleanup of temporary data. Technically, a SUSHI application is implemented in a single Ruby file as a single Ruby class that takes over the SUSHI application super class using the template method design pattern (see Additional file 6).

### SUSHI architecture follows meta-level design

SUSHI consists of three modules that are implemented in Ruby: 1) The SUSHI application module, as discussed above. 2) A Workflow Manager that performs job execution either on a local host or on a grid or cloud environment. 3) The SUSHI server that relies on Ruby on Rails and provides the web front-end. The SUSHI server delegates the core function of generating a job script to the SUSHI application, and the SUSHI application communicates with the Workflow Manager for job submission. SUSHI relies heavily on Ruby meta-programming in that the executable code is dynamically generated at run time. During the code execution, all graphical components are dynamically generated based on the SUSHI application. This process follows the basic principles of Ruby on Rails: DRY (Don’t Repeat Yourself) and CvC (Convention over Configuration). Namely, SUSHI application serves as the data source for the generation of the graphical components of the GUI. As a consequence every data analysis that is implemented as a SUSHI application is directly available in the web front-end of the SUSHI server. The implementation of a SUSHI application does not need any web development knowledge, so that the bioinformatician can focus on data analysis aspects.

### Installation

The SUSHI server is implemented in Ruby on Rails and can be installed in one step including all dependencies. The default installation uses the WEBrick web server application and SQLite3 database management system. Alternatively, SUSHI can be configured to run using an Apache web-server and a MySQL backend. For more details, please refer to README.rdoc in the source repository, https://github.com/uzh/sushi.

## Results and discussion

### Use case RNA-seq data analysis

A commonly used minimal workflow in RNA-seq data analysis consists of the steps:

1. Map Reads with STAR aligner [14].
2. Compute expression values with the featureCounts function (Rsubread [15]).
3. Detect differential expressed genes with edgeR [16].

**Fig. 3:**
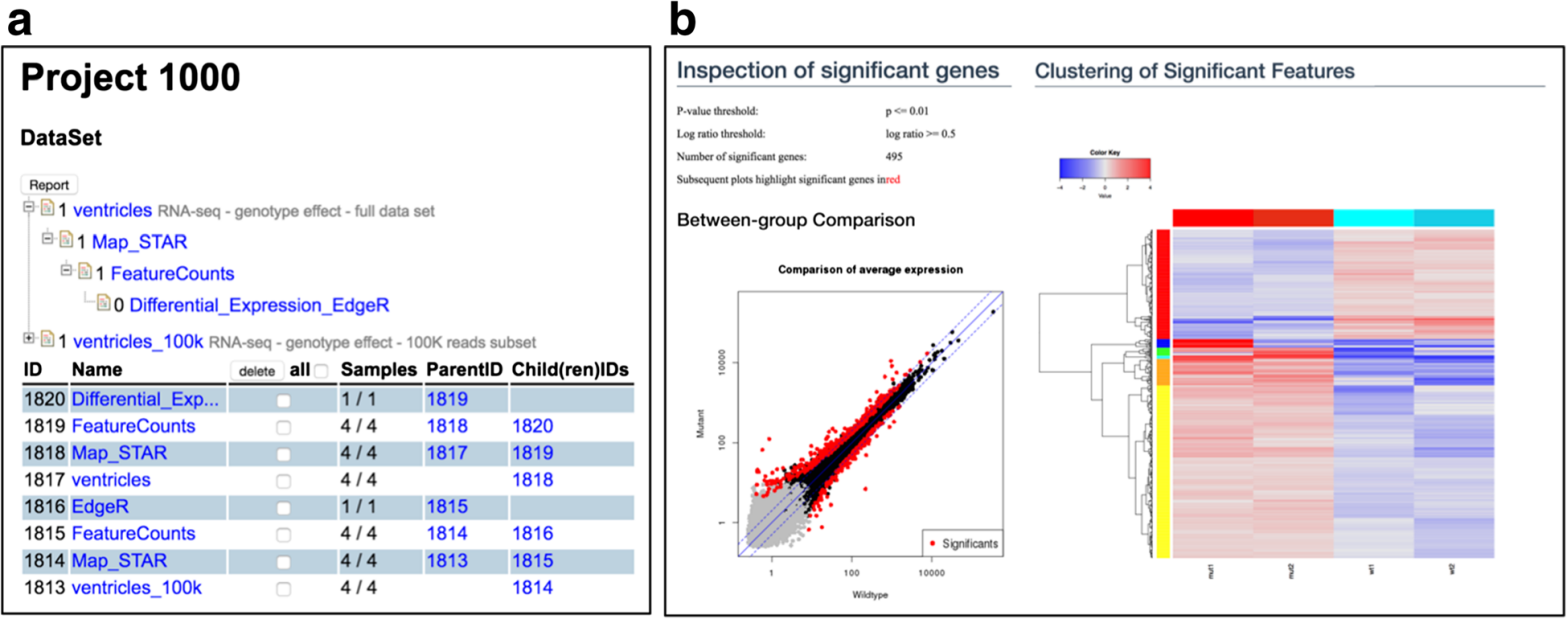
The screenshots of DataSet list and a part of a result generated by the edgeR SUSHI application. **a** The DataSets are listed with a tree view (top) and table view (bottom). In the tree view, each node indicates **a** DataSet and the parental node indicates the input DataSet for the child node. **b** Visualizations form the differential expression result the edgeR SUSHI application. We show a scatter plot with significantly differential expressed genes red-colored (left) and clustered heatmap (right). All calculated data is downloadable from this view

Figure 3a shows a representation of the generated datasets in SUSHI. The tree at the top indicates the hierarchical relationship and the bottom list shows the timeline of the generated data sets. Every data set is derived from its parent by running an application. Figure 3b shows visualizations that are generated by the last step, the assessment of differential expression between the wild-type and mutant samples in the data set. The scatter plot and the heat map indicate the significant genes and visualize the expression profiles. As with every SUSHI dataset, this result including all visualizations is downloadable and ready for offline use.

### Comparison with existing systems

We compare SUSHI to similar bioinformatics frameworks (Table 2 and Additional file 7). A main distinction of the various frameworks is whether they have a graphical user interface (GUI) or a command line interface (CLI). The majority of CLI systems is implemented as an extension of a programming language or implemented in a domain specific language (DSL), and targeted for bioinformaticians with programming skills. The GUI systems are implemented as web applications with a variety of components and functions. Their installation and configuration is more complex relative to CLI systems but their usage does not require programming skills, so that they are targeted towards biomedical researchers.

**Table 2:** Various types of workflow management systems are compared

System	UI	Language	Application	Meta-info.	Reproducibility	Documentation
Galaxy	GUI	Python	Workflow editor	Generating	Workflow	Galaxy file (.ga)
Chipster	GUI	Java	Workflow view	None	Workflow	Chipster file (.bsh)
GeneProf	GUI	Java	Workflow designer	None	Workflow	Image file
GenePattern	GUI,CLI	Java	Additional module	None	Pipeline	GenePattern library
Taverna	GUI,CLI	Java,Scufl	Plugin	Three types	Workflow	Workflow file
TOGGLE	CLI	Perl	Text file	None	Perl script	Text file
bpipe	CLI	Goovy,Java	bpipe script	None	bpipe script	bpipe script
NGSANE	CLI	Bash	Text file	None	trigger.sh	Text file
nestly	CLI	Python	Python script	None	nestrun	Python script
Snakemake	CLI	Python	Build file	None	snakemake	Build file
Ruffus	CLI	Python	Python script	None	Python script	Python script
Makeflow	CLI	C	Makeflow Language	None	Makeflow script	Workflow script
SUSHI	GUI,CLI	Ruby	Ruby script	tsv format	Shell script	Shell script

The systems are described by several features. The systems are categorized into two types by the user interface types, either GUI or CLI. Most systems have a proprietary format to save a workflow definition. More details are available in the Result section and in Additional file 7

Chipster [17] is designed as a GUI application and it does not have CLI or batch process mode, so that a user must run an application one by one manually. GenePattern [5] and Taverna [18]) provide both GUI and CLI. These systems are designed for GUI usage but provide also command-line access through special clients with an application programming interface (API). The SUSHI application model on the other side is designed for command line usage, and the web front-end is auto-generated using the Ruby on Rails meta-process.

In terms of reproducibility and documentation, CLI systems tend to have the entire analysis information in a single text file while GUI systems come with a relational database management system (RDBMS) that provides this and additional functionality. Generally, the framework itself is required to run an application or workflow again to reproduce results. Different from that SUSHI provides the inherent advantages of GUI systems but still generates a set of human-readable shell scripts file that contain all the processing information and can be run independent of SUSHI (see the scripts in Additional files 1 and 3). In essence, SUSHI conveniently ensures full documentation and high reproducibility but is not needed to reproduce the analysis results.

### Representation of meta Information

Accurate and high quality meta-information is necessary for the interoperability and integration of different data sources. However even 10 years after the minimal information about microarray experiment (MIAME) [19] guidelines have been established there is no consent how this should be implemented. This can be seen from the fact that different repositories and consortia use different implementations. Examples are the GEO SOFT [20], Sequence Read Archive (SRA) XML [21], ENCODE [22], modENCODE [23], and ISA-TAB [24]. Given this situation, we decided that SUSHI should not implement its own constraints on meta-information. It is up to the data providers to decide, for example, which meta-information fields are provided and whether they are filled with free text, controlled vocabularies or terms from an ontology. SUSHI simply makes sure that the meta-information is preserved and that the analysis results are linked to the source data and accompanying meta-information. It goes without saying that we always encourage to use controlled vocabularies and ontologies wherever possible.

### Productive use and user acceptance

**Fig. 4:**
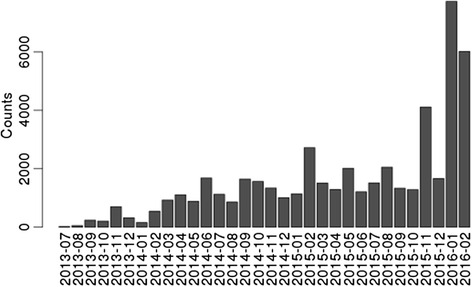
The number of submitted jobs using SUSHI at the Functional Genomics Center Zurich. It has been increasing since 2013 and now more than 5000 jobs are submitted on SUSHI at the Functional Genomics Center Zurich

SUSHI is in productive use at the Functional Genomics Center Zurich and has been used to analyze NGS data in more than 1000 projects. A publicly available instance is placed at http://fgcz-sushi-demo.uzh.ch. The productive instance is integrated with the project and data management system B-Fabric [25, 26] that handles projects, users, generated data, and access control. Currently we have implemented applications that support NGS analysis workflows for RNA-seq, small RNA-seq, ChIP-seq, *de novo* assembly, genotyping and variant analysis. The number of submitted jobs using SUSHI has been constantly increasing. Figure 4 shows the monthly submitted jobs at the Functional Genomics Center Zurich since 2013 which has reached now up to 5000 submitted jobs per month.

We have introduced SUSHI step-wise at the Functional Genomics Center Zurich. Initially, we supported only the most frequently used analyses such as RNA-seq and SNP calling, and subsequently we supported other applications like small RNA-seq, ChIP-seq and de novo assembly. SUSHI was readily adopted by the bioinformaticians because they had an immediate direct net benefit without a compromise on the flexibility in terms parameter choices. SUSHI development was driven and shaped by direct user feedback, which also broadened the acceptance.

### Advanced separation of SUSHI concerns

The separation of concerns (SoC) is a software design principle in computer science [27]. It is now widely accepted and adopted in a variety of computer systems and software design such as object oriented programming and modularity of software design. The Model-View-Controller (MVC) design pattern is a typical example of separation of concerns for better software maintainability. The meta-level design is one type of advanced separation of concerns (ASoC) beyond object-oriented which can be seen in recent software paradigms such as generic programming, generative programming, and meta-programming. For example, the reflection architecture in Pattern-Oriented Software Architecture (POSA) [28] separates a system into meta-level and base-level and by controlling a meta-level it triggers a change at the base-level that actually provides a service to a user.

The current situation of the common NGS data analysis, such that several independent software applications are combined and chained to produce a final result, presents the two following main aspects: 1) which applications are used, and 2) how they are actually used. SUSHI separates these aspects (*concerns*) into a meta-process with meta-information (SUSHI application and DataSet object) and a base-process (shell scripts that do the data analysis). This separation of concerns results in the loose coupling of SUSHI into its system level and user application level. This improves the independence of SUSHI applications and shell scripts.

## Conclusions

SUSHI targets both biologists and bioinformaticians as users. Analyzing data with SUSHI does not require programming skills, while adding new SUSHI applications requires only basic experience in writing scripts using the syntax of shell, R, Python, Ruby, or similar. SUSHI is particularly attractive to data analysis experts and bioinformaticians. SUSHI relieves users from administrative burdens and aids documentation and data organization. The full flexibility of the underlying tools stays untouched and can be directly accessed. Additionally, the fact that datasets are fully defined on the file system lets users prototype new workflows without the need to integrate those in the SUSHI instance. Finally, experts can automate data analysis tasks with the command line interface and are not limited to the web interface.

The design of the SUSHI system is driven by the idea of having analysis results fully defined and self-contained on the file system. In fact, if SUSHI is shut down, all the results can still be used and interpreted. All the meta-information is available in human readable tabular formats and all job scripts are contained with no back reference to the SUSHI framework. SUSHI provides no Laboratory Information Management System (LIMS) or computing functionality. Instead, through its open architecture it readily integrates with existing LIMS systems and computing resources.

In one solution, SUSHI provides at the same time fully documented, high level NGS analysis tools to biologists and an easy to administer, reproducible approach for large and complicated NGS data to bioinformaticians. This is mostly obtained by using the meta-level system design. Bioinformaticians will be freed from the boring tasks of managing software application and documentation and they can focus more on method development and on data analysis itself. The separation of the Workflow Manager from the SUSHI server makes the adaptation to any kind of computing facility easy and leaves the possibility to scale up.

The meta-level system design gives the simple but powerful framework of no data representation: SUSHI fully delegates the definition of dataset semantics to the user. The SUSHI system itself only defines how meta-information is used in a SUSHI application. It yields portability of datasets and lowers the barrier to reuse data and augment human readability of data set meta-information. The meta-level system design produces the decoupling of the meta-process from the base application process, which increases the degrees of freedom on the user side and contributes to the flexibility and scalability of the system.

## Availability and requirements

- Project name: SUSHI.
- Project homepage: https://github.com/uzh/sushi.

The demo installation is available at http://fgcz-sushi-demo.uzh.ch.

- Operating system(s): Platform independent but we recommend Unix-like system such as Ubuntu Linux and MacOS X.
- Programming language: Ruby (> = 1.9.3).
- Other requirements: Ruby on Rails (> = 3.2.9, < 4.0).
- License: MIT.

For the RubyGems library dependency, please refer to the Additional file 8, Gemfile.lock, which is also included in the Git repository.

## Abbreviations

API, application programming interface; ASoC, advanced separation of concerns; ChIP-seq, chromatin immunoprecipitation and next-generation DNA sequencing; CLI, command line interface; CvC, convention over configuration; DRY, don’t repeat yourself; DSL, domain specific language; GUI, graphical user interface; LIMS, laboratory information management system; MIAME, minimum information about a microarray experiment; MVC, model-view-controller; NGS, next generation sequencing; RDBMS, relational database management system; RNA-seq, RNA sequencing; SoC, separation of concerns; SRA, sequence read archive.

## Additional files

Additional file 1: An example of minimal SUSHI application to generate FastQC job script. (RB 658 bytes) Additional file 2: The result dataset produced by the FastQC SUSHI application written as Additional file 1. (TGZ 706 kb) Additional file 3: An example of SUSHI application to generate TopHat job script. (RB 2 kb) Additional file 4: The result dataset produced by the TopHat SUSHI application written as Additional file 3. (TGZ 2223 kb) Additional file 5: List of SUSHI application. (XLS 5 kb) Additional file 6: Class diagram of SUSHI application. (PNG 61 kb) Additional file 7: Comparison of workflow management systems. (XLS 35 kb) Additional file 8: List of required RubyGems library. It is bundled in the SUSHI source code as Gemfile.lock. (TXT 4 kb)
