# Supplementary figures and images for: SUSHI: an exquisite recipe for fully documented, reproducible and reusable NGS data analysis
Source: BMC Bioinformatics. 2016 Jun 2;17:228. doi: 10.1186/s12859-016-1104-8 (PMC4890512; doi:10.1186/s12859-016-1104-8)

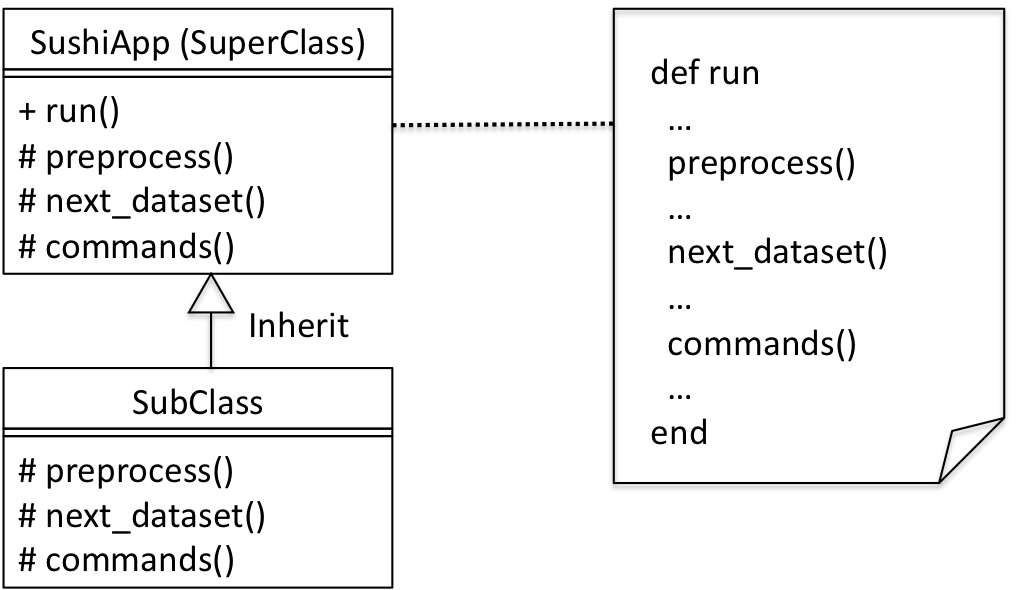

Supplement: Additional file 6: — Class diagram of SUSHI application. (PNG 61 kb) [file 12859_2016_1104_MOESM6_ESM.png]
