# Supplementary material for: SUSHI: an exquisite recipe for fully documented, reproducible and reusable NGS data analysis
Source: BMC Bioinformatics. 2016 Jun 2;17:228. doi: 10.1186/s12859-016-1104-8 (PMC4890512; doi:10.1186/s12859-016-1104-8)
Supplement: Additional file 2: — The result dataset produced by the FastQC SUSHI application written as Additional file 1. (TGZ 706 kb) [file 12859_2016_1104_MOESM2_ESM.tgz › FastqcMinimal_result_dataset/MutantSample_3_R1_fastqc.zip!/MutantSample_3_R1_fastqc/fastqc_report.html]

MutantSample\_3\_R1.fastq.gz FastQC Report 

FastQC Report

Tue 26 Apr 2016  
MutantSample\_3\_R1.fastq.gz

## Summary

- Basic Statistics
- Per base sequence quality
- Per tile sequence quality
- Per sequence quality scores
- Per base sequence content
- Per sequence GC content
- Per base N content
- Sequence Length Distribution
- Sequence Duplication Levels
- Overrepresented sequences
- Adapter Content
- Kmer Content

## Basic Statistics

| Measure | Value |
| --- | --- |
| Filename | MutantSample\_3\_R1.fastq.gz |
| File type | Conventional base calls |
| Encoding | Sanger / Illumina 1.9 |
| Total Sequences | 100000 |
| Sequences flagged as poor quality | 0 |
| Sequence length | 50 |
| %GC | 49 |

## Per base sequence quality

## Per tile sequence quality

## Per sequence quality scores

## Per base sequence content

## Per sequence GC content

## Per base N content

## Sequence Length Distribution

## Sequence Duplication Levels

## Overrepresented sequences

| Sequence | Count | Percentage | Possible Source |
| --- | --- | --- | --- |
| CTGCCAGTAGCATATGCTTGTCTCAAAGATTAAGCCATGCATGTCTAAGT | 879 | 0.8789999999999999 | No Hit |
| GTCGGCATGTATTAGCTCTAGAATTACCACAGTTATCCAAGTAGGAGAGG | 504 | 0.504 | No Hit |
| CCTGCCAGTAGCATATGCTTGTCTCAAAGATTAAGCCATGCATGTCTAAG | 311 | 0.311 | No Hit |
| TGCCAGTAGCATATGCTTGTCTCAAAGATTAAGCCATGCATGTCTAAGTA | 304 | 0.304 | No Hit |
| CCGTCGGCATGTATTAGCTCTAGAATTACCACAGTTATCCAAGTAGGAGA | 252 | 0.252 | No Hit |
| CCCGTCGGCATGTATTAGCTCTAGAATTACCACAGTTATCCAAGTAGGAG | 250 | 0.25 | No Hit |
| CGTCGGCATGTATTAGCTCTAGAATTACCACAGTTATCCAAGTAGGAGAG | 217 | 0.217 | No Hit |
| GGAGTCTAACGCGTGCGCGAGTCAGGGGCTCGTCCGAAAGCCGCCGTGGC | 201 | 0.201 | No Hit |
| GCCAGTAGCATATGCTTGTCTCAAAGATTAAGCCATGCATGTCTAAGTAC | 196 | 0.196 | No Hit |
| CGAACTCTCCAGAGGTGGCAACTGGCCTCAGACACCATGGCACCAAAGAA | 174 | 0.174 | No Hit |
| TCCTGCCAGTAGCATATGCTTGTCTCAAAGATTAAGCCATGCATGTCTAA | 112 | 0.11199999999999999 | No Hit |
| CTCACCCGGCCCGGACACGGACAGGATTGACAGATTGATAGCTCTTTCTC | 106 | 0.106 | No Hit |
| CGGGCGCTGACCCCCCTTCCCGGGGGGGGATGCGTGCATTTATCAGATCA | 104 | 0.104 | No Hit |
| CCTCACCCGGCCCGGACACGGACAGGATTGACAGATTGATAGCTCTTTCT | 103 | 0.10300000000000001 | No Hit |

## Adapter Content

Can't analyse adapters as read length is too short

## Kmer Content

| Sequence | Count | PValue | Obs/Exp Max | Max Obs/Exp Position |
| --- | --- | --- | --- | --- |
| GAGTCTA | 25 | 4.4035834E-5 | 44.01701 | 2 |
| TACGGTA | 25 | 4.416619E-5 | 43.995 | 44 |
| TACCTTG | 30 | 2.5064764E-6 | 43.995 | 9 |
| TTACGGT | 25 | 4.416619E-5 | 43.995 | 43 |
| AGGGAAC | 35 | 7.2293824E-6 | 37.71 | 42 |
| AGTCTAA | 30 | 1.289676E-4 | 36.68084 | 3 |
| CCTTACG | 30 | 1.2934812E-4 | 36.6625 | 41 |
| CTTACGG | 30 | 1.2934812E-4 | 36.6625 | 42 |
| CAGTTAC | 40 | 1.7987428E-5 | 33.012756 | 5 |
| GTTACCT | 40 | 1.8049173E-5 | 32.99625 | 7 |
| TTACCTT | 40 | 1.8049173E-5 | 32.99625 | 8 |
| ACCTTGT | 40 | 1.8049173E-5 | 32.99625 | 10 |
| TCCCTTA | 35 | 3.1990936E-4 | 31.425 | 39 |
| GGGAACC | 35 | 3.1990936E-4 | 31.425 | 43 |
| CCCTTAC | 35 | 3.1990936E-4 | 31.425 | 40 |
| GGAGTCT | 50 | 2.2898312E-6 | 30.873684 | 1 |
| AGTTACC | 45 | 4.0221326E-5 | 29.344673 | 6 |
| CTTGTTT | 45 | 4.0358995E-5 | 29.33 | 12 |
| AAGTTGG | 30 | 0.0057233507 | 29.33 | 43 |
| AAAGTTC | 40 | 6.9914607E-4 | 27.496876 | 22 |

Produced by FastQC (version 0.11.3)
